# Caloric restriction maintains OX40 agonist-mediated tumor immunity and CD4 T cell priming during aging

**DOI:** 10.1007/s00262-014-1542-y

**Published:** 2014-03-30

**Authors:** Michelle Farazi, Justine Nguyen, Josef Goldufsky, Stephanie Linnane, Lisa Lukaesko, Andrew D. Weinberg, Carl E. Ruby

**Affiliations:** 1 Department of General Surgery, Rush University Medical Center, 1653 W. Congress Pkwy., Rm JS806, Chicago, IL 60612 USA; 2 Department of Immunology/Microbiology, Rush University Medical Center, Chicago, IL 60612 USA; 3 Cancer Center, Rush University Medical Center, Chicago, IL 60612 USA; 4 Providence Portland Medical Center, Earle A Chiles Research Institute, Portland, OR 97213 USA

**Keywords:** Aging, Caloric restriction, Resveratrol, T cell, Immunotherapy

## Abstract

Immune responses wane during aging, posing challenges to the potential effectiveness of cancer immunotherapies. We previously demonstrated that in the context of a promising immunotherapeutic, OX40 agonist (αOX40), older animals exhibited impaired anti-tumor immune responses and diminished CD4 T cell effector differentiation. In this study, we hypothesized that tumor immune responses could be maintained during aging through caloric restriction (CR) or dietary supplementation with resveratrol (RES), a CR mimetic. Mice were placed on either a calorically restricted diet or a RES-formulated diet starting between 4 and 6 months of age and continued until mice reached 12 months of age. Tumor immune responses were assessed after challenging with either sarcoma or breast tumor cells followed by αOX40 treatment. Our results show that CR, but not RES, maintained OX40-mediated anti-tumor immunity. In addition, CR fully sustained antigen-specific CD4 T cell priming in aged hosts (12 months old), whereas tumor-specific CD8 T cell priming was not fully maintained compared to young reference animals (2 months old). Thus, CR appears to maintain immunological fitness of the CD4 T cell priming environment during aging, which is critical for optimal OX40-mediated responses.

**Electronic supplementary material:**

The online version of this article (doi:10.1007/s00262-014-1542-y) contains supplementary material, which is available to authorized users.

## Introduction

It is well understood that the immune system recognizes and eradicates tumor cells. More recently, immune-based therapies have been shown to be effective in mediating durable responses in cancer patients with advanced disease. In fact, several immunotherapies, such as high-dose IL-2, CTLA-4 blockade and an autologous dendritic cell-based tumor vaccine, have been shown to produce significant benefit to a subset of patients. Additional cancer immunotherapeutic approaches, including the blockade of the co-inhibitory molecules PD-1 and adoptive T cell therapy, have produced objective clinical responses and in some cases produced complete responders in clinical trials [1, 2]. However, clinical success of established and developing immunotherapies could ultimately be dependent on the effect of aging on immunity, as most cancer patients are elderly.

Age-related changes occur in both innate and adaptive immunity to induce a state of immune deficiency that can have an impact on tumor immune responses. It has been documented that aging compromises T cell function, including the reduction in circulating naïve T cells, impairment of proliferation, and a decrease in IL-2 production [3–5]. Innate cells, such as macrophages, neutrophils and natural killer cells, can undergo age-mediated decreases in function [6]. Dendritic cells (DCs), which are critical for the priming of adaptive immune responses, from aged animals have been shown to be dysfunctional and are reduced in number compared with younger animals [7]. The immunological decline associated with aging coincides with an increased incidence of tumors in older individuals (>65 years old) [8, 9]. Although a direct link between age-impaired immune function and tumor outgrowth has not been fully established, tumor immune responses induced by vaccines or other immune-based therapies have been shown to be deficient in older animals. For example, vaccines against breast tumors were significantly less effective in older animals [10]. In addition, we have published studies that described a dramatic age-related decrease in anti-tumor responses mediated by a promising immunotherapy, an OX40 agonist (αOX40) that stimulates the co-stimulatory TNF receptor CD134 [11, 12]. Our results suggest that a significant decrease in immune function within aged tumor-bearing hosts can result in diminished tumor rejection mediated by an immunotherapy.

Strategies to rejuvenate or maintain anti-tumor immunity during aging could improve immunotherapeutic outcomes in elderly cancer patients. At this time, caloric restriction (CR) is the most reliable approach to maintain immune function during aging. CR is a substantial reduction in caloric intake (30–60 % below ad libitum levels), resulting in animals that have fewer and less severe immunological deficiencies at advanced ages compared to ad libitum controls [13]. CR can maintain immune cell function, as seen in readouts such as T cell proliferative capacity and IL-2 production. These cellular changes may account for the significant increase in protection against influenza infection observed in aged CR mice [14–16]. In addition to rodents, CR studies with non-human primates also revealed similar immunological effects, suggesting that humans would likely respond favorably to CR [17].

Maintaining CR at levels shown to be beneficial is extremely difficult lending interest in compounds able to recapitulate the effects of CR. One compound of particular interest is the plant phytoalexin resveratrol (RES), which has received significant attention as a multi-targeted anti-aging agent [18]. This compound has been shown to mediate similar physiological, biological, and molecular effects of CR in models ranging from *C. elegans* to more complex non-human primate models [19, 20]. RES can delay the onset of a number of diseases though activation of multiple pathways, specifically the NAD^+^/SIRT pathway, and increase mitochondrial function [21]. In addition, RES has been shown to be an effective activator of SIRT1, but the exact mechanism of SIRT1 activation has not been fully elucidated [19]. Despite these promising features, pharmacokinetic studies have demonstrated that RES is readily metabolized, prompting its low bioavailability [19]. It is currently unknown whether RES or its metabolites are able to accumulate at high enough concentrations in the tissues to be effective in vivo. In addition, it is unclear if RES does in fact act directly through SIRT proteins or through other targets, and whether the results of numerous in vitro studies demonstrating protective effects can be recapitulated in vivo.

In the studies presented here, we sought to determine whether tumor immune responses mediated by an immunotherapy could be maintained during aging by dietary interventions, CR and/or RES supplementation. Mice were subjected to CR or RES supplementation starting at ages when the immune response was observed to be highly responsive and continued until the mice reached 12 months of age, an age when immune responses were shown to be significantly diminished [11]. These mice were then challenged with either one of two different tumors that were demonstrated to be responsive to αOX40-mediated tumor immunity in young mice, but were unresponsive in 12-month-old as well as elderly 20-month-old mice. Additional mice were also immunized with a protein antigen (ovalbumin) in the context of αOX40 treatment. It was observed that CR increased OX40-mediated anti-tumor immunity in both 12-month-old and 18–24-month-old mice compared with age-matched ad libitum controls. In contrast, RES supplementation failed to have a significant effect on tumor immunity in aged mice. Analysis of the T cell component of the immune response in the mice following dietary intervention suggested that OX40-enhanced CD4 T cell activation was maintained in aged CR mice compared to controls. Interestingly, CD8 T cell responses were largely unaffected. These findings demonstrate CR can maintain critical immune responses following treatment with a cancer immunotherapeutic.

## Materials and methods

### Mice and diets

Six-week-old female Balb/c and C57BL/6 mice were purchased from Harlan Laboratory and used at 8 weeks of age. For the aged mice, 9–10-month-old mice were purchased from Harlan and used at about 12 months of age, and 12-, 18-, and 24-month-old ad libitum mice from the National Institute on Aging (NIA). Calorically restricted female C57Bl/6 mice (mean age = 12, 18 and 24 months) were purchased from the NIA. DO11.10, OTII, and OTI mice were bred and housed until reaching a mean age of about 2 months of age. Six-month-old female C57Bl/6 and Balb/c mice were fed either a common laboratory rodent diet (LRD) (NIH31, Harlan, IN; or LabDiet5001, PharmaServ, MA), a purified diet (PD) (AIN93M, Harlan/Teklad, WI), or a RES supplemented diet (AIN93M/100 ppm RES, Harlan/Teklad, WI). RES and pterostilbene were purchased from Cayman Chemical (Ann Arbor, MI) and formulated by Harlan/TekLad (WI). The formulated diets were vacuumed, packed, and stored at 4 °C (up to 12 months). The AIN93M is formulated for the maintenance of rodents [22] and is comprised of less protein (12.4 vs 23.9 %) and fat (4.1 vs 5.7 %) compared the LRD, LabDiet5001. Once mice on formulated diets reached 12 months of age, they were placed on the PD for 7–10 days prior to experimentation. All the animals had free access to water and their experimental food throughout the experiment. All mice were maintained at the Rush Comparative Research (CRC) animal facility, and all animal studies were approved by the Rush University IACUC committee.

### Tumor models

MCA205 (H12) is derived from 3-methylcholanthrene-induced mice and can be passaged in culture. These cells were suspended in PBS and 0.3–1.0 × 10^6^ injected s.c. into C57BL/6 mice. The variant MCA205-OVA tumor cell line transgenically expresses the egg protein ovalbumin, as a surrogate antigen. EMT-6 tumor cells, derived from a mouse mammary carcinoma, were suspended in PBS and 1 × 10^5^ injected into the flanks of female Balb/c mice. Depending on the experiment, mice were then injected i.p. with 200–250 μg αOX40 (OX86) or rat IgG, 3 and 7 days following tumor challenge or 4, 6, and 10 days after tumor challenge. Mice were then monitored for tumor growth and killed if the tumor became ulcerated or growth reached 150 mm^2^.

### DO11.10, OTII, and OTI transgenic TCR adoptive transfer experiments

DO11.10 and OTII transgenic TCR CD4 T cells are specific to a peptide of the ovalbumin protein (a.a. 323–339) and can be identified by the antibody designated KJ-126 or the congenic marker thy1.1 or Vα2 and Vβ5. OTI transgenic TCR CD8 T cells, specific to a peptide of the ovalbumin protein (a.a. 257–264), were identified by the congenic marker thy1.1 or Vα2 and Vβ5. Spleens and LNs were harvested and processed by crushing between two frosted glass microscope slides and red blood cells lysed with ACK (Lonza, CA). The frequency and number of transgenic T cells were identified by FACS analysis prior to transfer. A total of 1 × 10^6^ transgenic TCR T cells were adoptively transferred i.v. into Balb/c or C57Bl/6 recipients. One day later, mice were immunized s.c. with 500 μg ovalbumin (Sigma, St. Louis, MO) and 50 μg of OX40 agonist (OX86) or IgG control (Jackson ImmunoResearch, PA). The following day, mice were given a second injection of αOX40 or rat IgG. For experiments in the tumor setting, MCA205-OVA tumor cells (1 × 10^6^) were transplanted 1 day after adoptive transfer of 1 × 10^6^ transgenic TCR OTII or OTI T cells. One day after tumor transplantation, mice were injected with 200 μg of αOX40 or IgG control i.p. and a total of 7 days after tumor transplantation mice were killed.

### FACS analysis of T cells from antigen-draining lymph nodes

T cells were liberated from antigen-draining LNs by disruption between two frosted glass slides and counted using a hemacytometer. Cells were stained with various combinations of the following fluorescently labeled antibodies: PerCP Cy5.5.-CD4, APC-CD25, PE Cy7-CD8, PE-CD25, APC-KJ-126, FITC Thy1.1, FITC Vβ5, APC Vα2, e450-CD62L, and biotinylated-KJ-126 (eBioscience, CA). Harvested samples, isotype controls, and single stain controls were run on the FACSCanto (Becton–Dickinson, NJ).

### Intracellular cytokine staining

T cells from the antigen draining lymph nodes and contralateral lymph nodes were harvested from DO11.10, OTII, or OTI adoptively transferred recipients 4 or 7 days after challenge and stimulated for at least 6 h in vitro with 5 μg/ml ovalbumin_323–339_ (DO11.10 and OTII) or ovalbumin_257–264_ (OTI) and 1 μg/mL Golgi stop (BD Biosciences Pharmingen, CA). Cells were harvested and stained with PerCP Cy5.5-CD4, APC-KJ-126, FITC Thy1.1, FITC Vβ5, APC Vα2, or APC Cy7-CD8 (eBioscience, CA). Cells were made permeable with CytoPerm/PermWash buffers and stained with PE-Cy7-IFNγ (BD Biosciences Pharmingen, CA). T cells were analyzed on the BD FACSCanto (Becton–Dickinson, NJ).

### Statistical analysis

For all experiments, Student’s *t* test (two-tailed) was used to compare means of selected groups. For analysis, values of *p* ≤ 0.05 were considered significant and expressed as follows: *p ≤ 0.05 and **p ≤ 0.001 if not specifically stated.

## Results

### Caloric restriction improves the ability of an OX40-targeted immune therapy to mediate tumor free survival in aged tumor-bearing mice

**Fig. 1:**
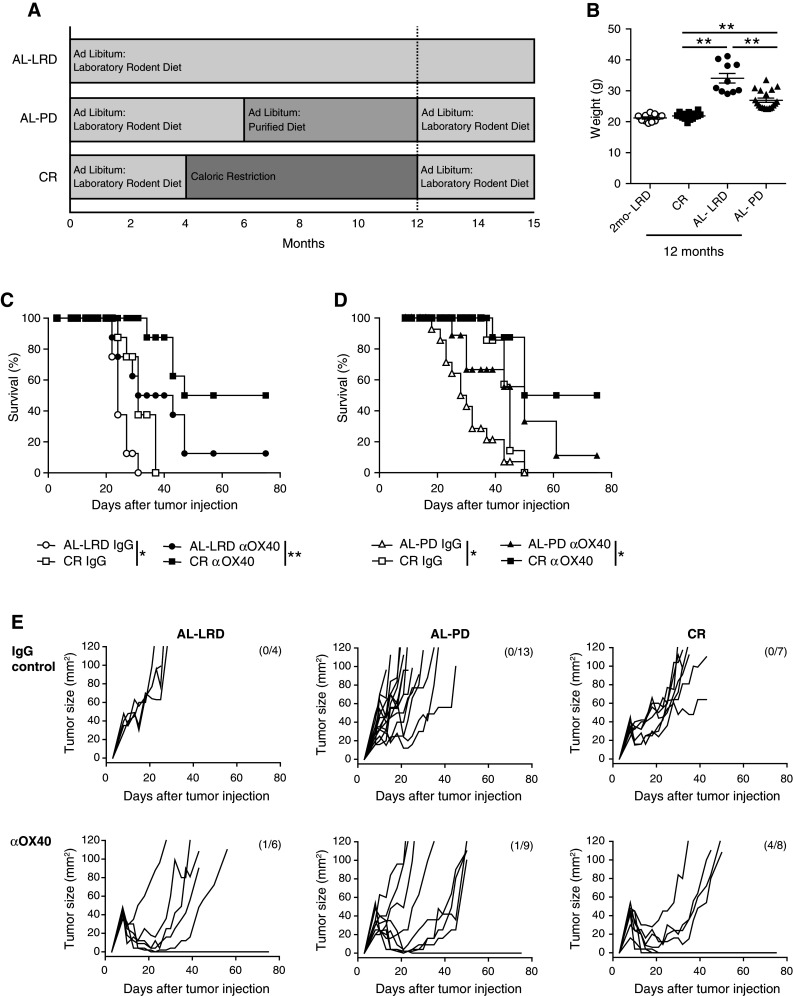
Agonist OX40-mediated tumor free survival was greater in calorically restricted 12-month-old mice compared to ad libitum controls. **a** Dietary protocol; **b** 12-month-old female C57Bl/6 mice on a caloric restricted diet (CR), an ad libitum laboratory rodent diet (AL-LRD) or an AL-PD were weighed 1 day prior to tumor challenge and compared to 2-month-old mice; **c** 12-month-old AL-LRD mice; or **d** 12-month of AL-PD mice and 12-month-old (CR) were injected s.c. with 3 × 10^5^ MCA205 tumor cells. Three and seven days later, αOX40 or rat IgG (200 μg) was injected i.p. and mice were assessed for survival (Kaplan–Meier). *Data* represent one of two independent experiments with *n* = 4–13 mice per treatment group; **e** Tumor growth of individual mice

Our previous studies demonstrated the ability of αOX40 treatment to be effective in improving tumor free survival in young tumor-bearing mice, but the same effect was not observed in older 12-month or 20-month-old tumor-bearing mice [11]. Using the same MCA205 sarcoma tumor cell line, we sought to determine whether CR would maintain effective αOX40-mediated anti-tumor immune responses in aged 12-month-old mice. CR mice (mean age of 12 months) were purchased from the NIA. These mice were initially placed on a 40 % restricted diet (NIH31/NIA Fortified) at a young age (16 weeks) and maintained as outlined (www.nia.gov). Calorically restricted mice were weighed and compared to (1) 2-month-old mice, (2) 12-month-old mice that were on an ad libitum laboratory rodent diet (AL-LRD) (e.g., NIA31), and (3) mice that were on an AL-LRD for 6 months followed by an ad libitum purified diet (AL-PD) until 12 months of age (Fig. 1a). The purified diet used, AIN93M, is comprised of less protein (12.4 vs 18.0 %) and fat (4.1 vs 4.7 %) when compared to a LRD, such as NIH31. Previous administration of the purified diet (AL-PD) appeared to reduce the weight of the mice compared to normal rodent diet (AL-LRD). The PD was initiated at 6 months of age, as this was a time when the αOX40-mediated anti-tumor immune response was intact and similar to the response in 2-month-old mice [11]. The weights of the aged dietary groups were significantly different, with the CR group being the lightest and the AL-LRD being the heaviest (Fig. 1b). Prior to tumor challenge, the calorically restricted mice were placed on an ad libitum diet, as tumor growth could be slowed by calorie restriction alone. The aged mice were then injected s.c. on their right flanks with MCA205 tumor cells followed by i.p. injections of αOX40 or rat IgG 3 and 7 days later as previously described [11]. Ad libitum (AL-LRD and AL-PD) mice and CR mice treated with rat IgG failed to reject the tumors, and the tumors grew out rapidly (Fig. 1c, d, e). Interestingly, in all experiments IgG control treated CR mice experienced a significant increase in survival compared to ad libitum mice (Kaplan–Meier). Mice (12 months old) from both ad libitum groups (AL-LRD and AL-PD) treated with αOX40 had dramatically decreased tumor free survival when compared with the αOX40 treated caloric restricted (CR) group of mice (Fig. 1c, d).

**Fig. 2:**
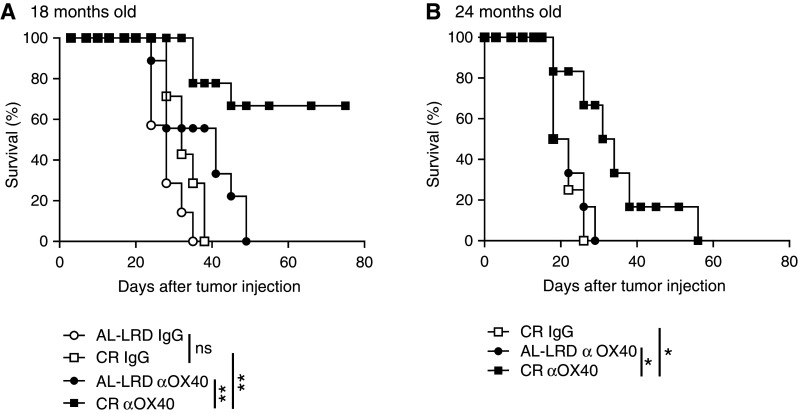
Agonist OX40-mediated tumor survival was greater in calorically restricted elderly mice compared to ad libitum controls. **a** 18- or **b** 24-month-old female C57Bl/6 mice on a caloric restricted diet (CR) or an ad libitum laboratory rodent diet (AL-LRD) were challenged (s.c.) with 3 × 10^5^ MCA205 tumor cells. Three and seven days later, αOX40 or rat IgG (200 μg) was injected i.p. and mice were assessed for survival (Kaplan–Meier).* Data* represent two independent experiments with *n* = 6–9 mice per treatment group

In a second set of experiments, we extended our study of CR on αOX40-mediated anti-tumor responses to include elderly mice. The mice aged 18 and 24 months of age (CR and AL-LRD) were challenged with MCA205 and treated with αOX40 and IgG controls like previously described. Tumor survival of the αOX40-treated CR mice was significantly greater than control AL-LRD tumor-bearing mice 18 or 24 month of age (Fig. 2). These findings indicate that long-term CR can enhance αOX40-mediated anti-tumor responses in 12-, 18- and 24-month-old mice.

### Neither resveratrol nor pterostilbene were able to significantly boost αOX40-mediated tumor immunity in aged tumor-bearing mice

**Fig. 3:**
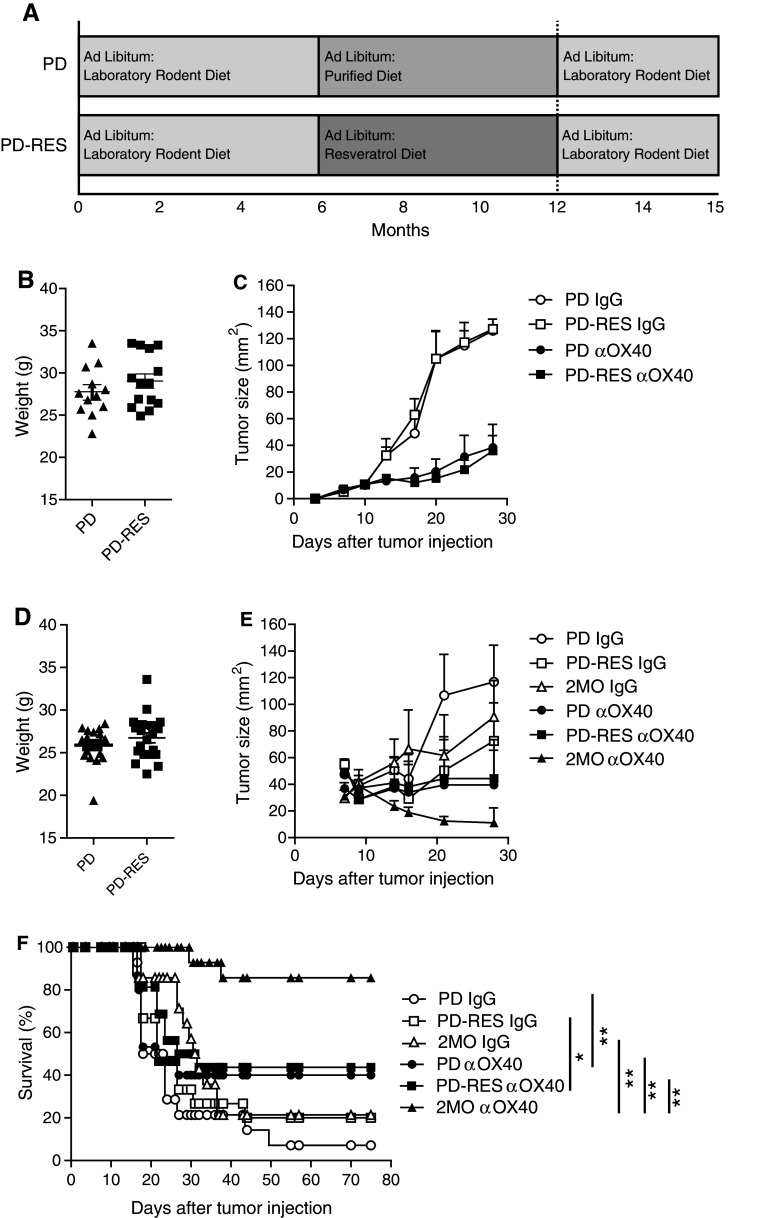
Long-term dietary supplementation with resveratrol did not significantly increase αOX40-mediated tumor free survival in aged mice. **a** Dietary protocol; **b** 12-month-old female C57Bl/6 mice on a purified diet (PD) or a purified diet supplemented with 1 mg/kg resveratrol (PD-RES) were weighed 1 day prior to tumor challenge; **c** Resveratrol (PD-RES) and purified diet (PD) fed mice (12 months old) were injected s.c. with 3 × 10^5^ MCA205 tumor cells. Three and seven days later, 200 μg αOX40 or rat IgG was injected (i.p.) and mice were monitored for tumor growth. *Data* are represented as a mean (±SEM) of one experiment (*n* = 10 per treatment group); **d** 12-month-old female Balb/c mice on a purified diet (PD) or a purified diet supplemented with 1 mg/kg resveratrol (PD-RES) were weighed 1 day prior to tumor challenge. Resveratrol and purified diet fed mice (12 months old) were injected s.c. with 1 × 10^5^ EMT6 tumor cells. Three and seven days later 200 μg αOX40 or rat IgG were injected i.p. and mice were monitored for tumor growth; (**e**) and survival (Kaplan–Meier); (**f**). *Quantitative date* are represented as means (±SEM) from one of three independent experiments (*n* = 8–10 mice per treatment group)

The major drawback with CR interventions for the broad prevention of human diseases is the difficulty of initiating and sustaining the 30–60 % restriction of caloric intake needed for beneficial effects. An alternative to CR may reside in compounds that mimic CR, such as resveratrol (RES). Unfortunately, the effects of RES on immunity are complex, conflicting, and largely untested in regard to aging and tumor immunity [23–26]. Thus, to determine whether RES maintains beneficial tumor immunity, mice were fed a diet supplemented with RES. A commonly used purified diet was supplemented with RES (100 ppm) to produce an estimated daily dose of 12.5 mg/kg for a normal mouse. This dose is based on a report that showed long-term ingestion of RES at this dose was well tolerated and closely mimicked the effects of CR on biological system maintenance in mice [27]. Interestingly, RES, at doses ranging as low as 100 ng/kg and as high as 1,500 mg/kg, can also induce biological effects [19, 20]. The formulated resveratrol diet (PD-RES) or the unsupplemented PD was introduced when mice were six months old and concluded when they reached 12 months of age (Fig. 3a). At this time, the mice were weighed (Fig. 3b, d) and placed on the LRD, as RES has been shown to alter tumor growth [28]. The 12-month-old mice (PD-RES and PD) were challenged s.c. with MCA205 tumor cells treated with αOX40 or rat IgG 3 and 7 days after tumor injection and tumor size measured (Fig. 3c). Dietary supplementation with RES failed to improve the αOX40-mediated anti-tumor immune responses in 12-month-old mice compared to control animals (PD). In addition, we assessed Balb/c mice placed on the same PD-RES and PD diets and challenged with the immunogenic tumor cell line EMT6. The αOX40 treatment induced significant tumor free survival in 2-month-old mice compared to rat IgG controls. However, there was no difference in tumor-free survival and tumor growth between 12-month-old resveratrol (PD-RES) fed mice and control diet (PD) fed mice, with both 12-month-old groups experiencing a dramatic decrease in survival compared to the αOX40-treated 2-month-old mice (Fig. 3e, f). These results indicate that dietary RES intake is unable to maintain effective anti-tumor immune responses in the context of αOX40 treatment.

In addition, the compound pterostilbene, a derivative of RES, has also been shown to reduce the affect of aging [29, 30] and has higher biological activity and greater bioavailability than RES [31] was also tested. This compound represents another dietary supplement, similar to RES, with the potential to positively affect tumor immune responses during aging. Purified diet was supplemented with pterostilbene (100 ppm), which correlated to the dose of RES previously used. The formulated pterostilbene diet (PD–PT) or the PD was introduced when mice were six months old and concluded when they reached 12 months of age. At 12 months, the mice on the pterostilbene diet were then placed on the PD, and all mice were challenged with EMT6 tumor and treated with αOX40 as described above. We observed no significant difference in OX40-mediated tumor-free survival between the pterostilbene fed mice and controls (Supplemental Figure 1).

### Caloric restriction maintains antigen-specific CD4 T cell priming and activation in both the tumor setting and following protein immunization in aged mice

Both T cells and immune cells of the priming environment have been shown to be affected during aging; however, age-related deficiencies in the priming environment appear to significantly contribute to immune dysfunction [32, 33]. Our previous findings demonstrate that young T cells undergo impaired T cell activation when primed in an older host and interestingly older T cells primed in a young host were not affected [11]. Furthermore, antigen-specific tumor immunity was significantly reduced in aged tumor-bearing mice, likely due to defects in the aged priming environment (Supplemental Figure 2). In these experiments, 2- and 12-month-old mice received ovalbumin-specific CD4 T cells (2 or 12 months old) and were then challenged with a tumor cell line that expressed the surrogate antigen OVA. Growth of this tumor, even though it expresses ovalbumin, is not significantly affected by αOX40 treatment in the absence of adoptively transferred T cells (data not shown). However, in adoptively transferred 2-month-old mice, the tumors experienced a significant decrease in size following αOX40 treatment, regardless of the age of transferred T cells. In contrast, the tumors in all the older mice steadily increased in size (Supplemental Figure 2). Thus, age-related deficiencies within the host environment could be the primary driver of impaired αOX40-mediated tumor regression.

**Fig. 4:**
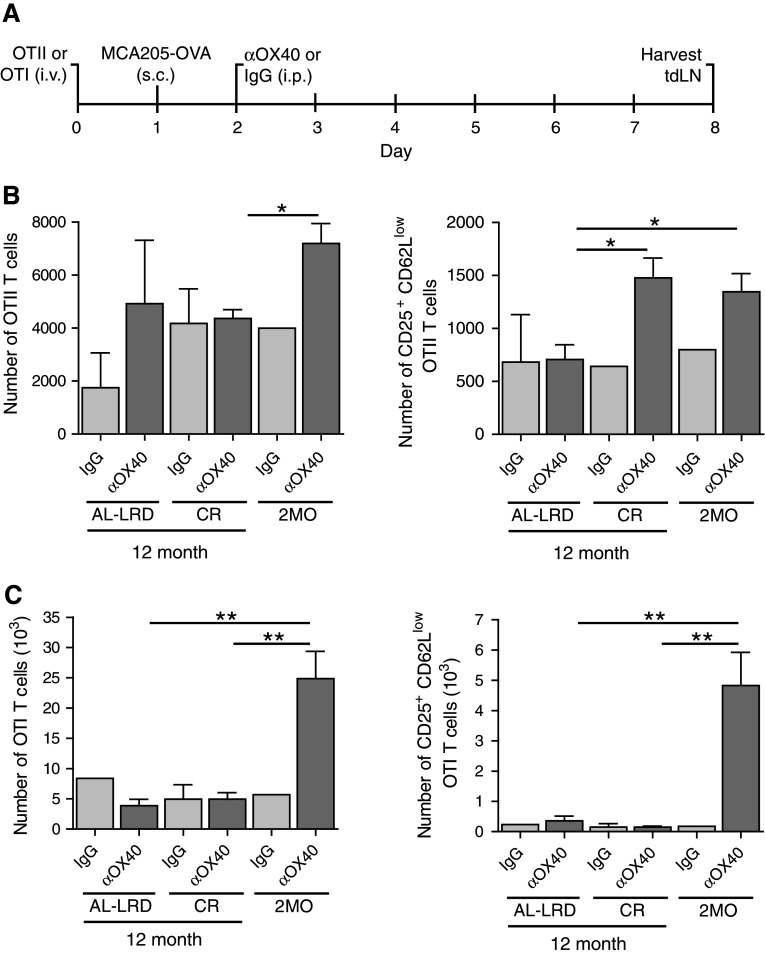
Caloric restriction maintained priming of tumor antigen-specific CD4 T cells. **a** Schematic of experimental design. Specifically, 2-month-old (2MO), 12-month-old AL-LRD and CR mice were adoptively transferred with 1 × 10^6^ OTII CD4 or 1 × 10^6^ OTI CD8 transgenic TCR T cells from a 2-month-old donor i.v. One day later, mice were transplanted s.c. with 1 × 10^6^ ovalbumin-expressing MCA205 tumor cells (MCA205-OVA). One day after tumor transplantation, mice were injected i.p. with 200 μg αOX40 or rat IgG. A total of 7 days after tumor transplantation, the tumor-draining LN (tdLN) was harvested, **b** Numbers of total and activated OTII CD4 cells were determined by FACs analysis of CD25 and CD62L expression, **c** Numbers of total and activated OTI CD8 cells were determined by FACs analysis of CD25 and CD62L expression. *Quantitative date* are represented as means (±SEM) from one of two independent experiments (*n* = 3–5 mice per treatment group)

We sought to determine whether CR would maintain the ability of the host priming environment to activate tumor antigen-specific T cells during aging. The priming and activation of both CD4 and CD8 T cells have shown to be required for effective OX40-mediated tumor immunity, as depletion of these cells abrogated the regression of tumors [34]; therefore, we assessed both antigen-specific CD4 and CD8 T cells. To avoid potential T cell intrinsic age-related deficiencies, young (2-month-old) ovalbumin-specific transgenic TCR CD4 T cells (OTII) or CD8 T cells (OTI) were adoptively transferred to young 2-month-old mice (2MO) or 12-month-old ad libitum mice (AL-LRD) or CR mice (CR). The mice were then challenged s.c. with ovalbumin-expressing MCA205 tumors. One day after, tumor challenge mice were injected with αOX40 or rat IgG and the draining LNs harvested 7 days later (Fig. 4a). The number of cells found in the draining LNs was not significantly different between the dietary groups (data not shown). Total numbers of transgenic T cells were enumerated and the number of activated antigen-specific T cells, CD25^+^ and CD62L^low^, were calculated. The number of activated OTII T cells was higher in CR mice compared to ad libitum mice (AL-LRD) and similar to those seen in the reference 2-month-old mice (2MO), reflecting an increase in the frequency of this population (Fig. 4b). In contrast, the number of antigen-activated OTI CD8 T cells from CR mice was similar to ad libitum mice (AL-LRD) and significantly less than young mice (2MO) (Fig. 4c). These data suggest CR maintains tumor-antigen-specific CD4 T cell priming to levels similar to young mice, but not CD8 T cell priming in the context of αOX40 treatment.

**Fig. 5:**
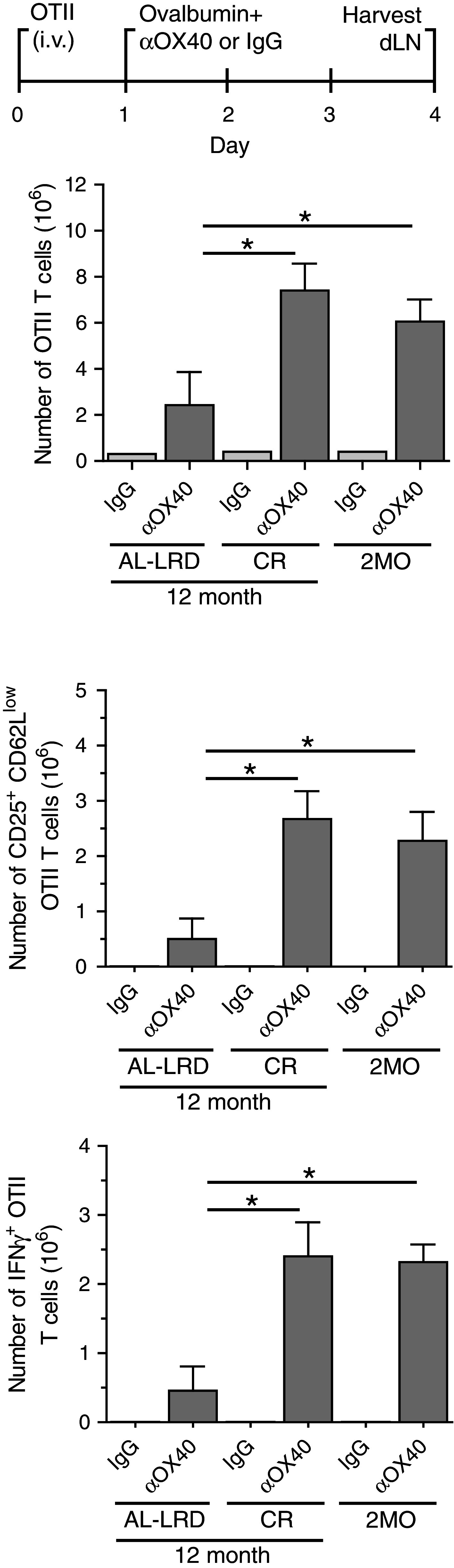
Antigen-specific T cell responses were maintained in calorically restricted aged mice. 2-month-old, 12-month-old AL-LRD and 12-month-old CR female C57Bl/6 mice were injected i.v. with 1 × 10^6^ OTII CD4 T cells T cells from 2-month-old donors. One day later, mice were injected s.c. with 500 μg ovalbumin and 50 μg of αOX40 or rat IgG. Four days after antigen challenge, the draining LNs were harvested and the antigen-specific T cells were enumerated. OTII cells were assessed for the expression of the activation markers CD25 and CD62L and the production of IFNγ after overnight restimulation with OVA peptide (a.a. 323–339). Quantitative date are represented as means (±SEM) from one of three independent experiments (*n* = 3–5 mice per treatment group)

To verify our previous findings, we assessed the affect of CR on the priming environment in an antigen-specific T cell model following s.c. immunization with protein antigen. Young (2-month old) ovalbumin-specific OTII CD4 T cells were adoptively transferred to young 2-month-old mice (2MO) as a reference or 12-month-old ad libitum (AL-LRD) or CR mice. Adoptively transferred mice were then challenged with whole ovalbumin protein and αOX40 or rat IgG. Four days after challenge, the draining LNs were harvested and the T cells assessed for activation and effector function. The number of activated (CD25^+^ CD62L^low^) and IFNγ-producing OTII CD4 T cells was significantly greater in the 12-month-old CR mice than 12-month-old ad libitum mice (AL-LRD) and was comparable with young 2-month-old mice (2MO) (Fig. 5), verifying the findings from the tumor setting. Thus, a second related antigen-specific T cell model adds additional evidence that CR maintains antigen-specific CD4 priming in the context of αOX40 treatment.

**Fig. 6:**
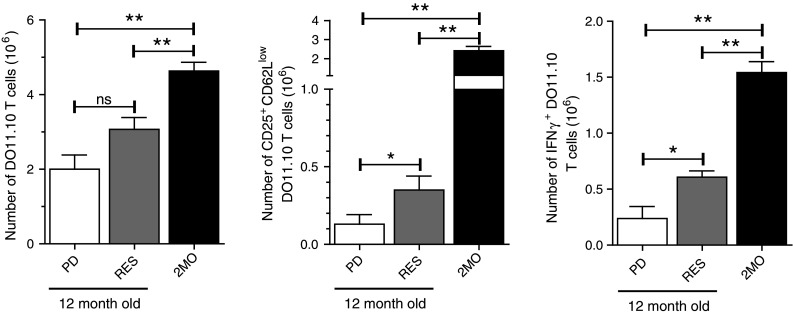
Resveratrol dietary supplementation and antigen-specific CD4 T cell responses in aged hosts. Six-month-old female Balb/c mice were placed on a purified diet (PD) or a 1 mg/kg resveratrol diet (RES) for 6 months. At 12 months of age, mice were returned to normal rodent chow diets and 2-month-old DO11.10 cells (1 × 10^6^) transferred into the aged mice and 2-month-old wt mice. One day later mice were injected s.c. with 500 μg ovalbumin and 50 μg of αOX40. Four days after Ag challenge, draining LNs were harvested and the antigen-specific T cells were enumerated and assessed for the expression of the activation markers CD25 and CD62L and the production of IFNγ after overnight restimulation with OVA peptide (CD4 a.a. 323–339). *Quantitative date* are represented as means (±SEM) from one of two independent experiments (*n* = 4 mice per treatment group)

Finally, the failure of RES to enhance αOX40-mediated tumor free survival in aged mice compared to aged control mice could be due in part to the inability of RES to maintain effective CD4 T cell priming. To test this, young (2 months old) ovalbumin-specific DO11.10 CD4 T cells were adoptively transferred to young 2-month-old mice or 12-month-old control diet fed mice (PD) or 12-month-old PD-RES mice. The mice were then challenged with ovalbumin protein and αOX40 or rat IgG, as previously described. Four days later, the number and frequency of activated (CD25^+^ CD62L^low^) and IFNγ-producing DO11.10 CD4 T cells in the draining LNs were assessed. The absolute numbers of DO11.10 T cells from the antigen-draining LNs of the αOX40-treated PD-RES mice were not significantly different from those harvested from the PD control mice also treated with αOX40 (Fig. 6). However, the numbers of activate (CD25^+^ CD62L^low^) and IFNγ^+^ cells were significantly greater in the aged αOX40-treated RES mice and control PD mice, but remained dramatically less than levels observed in the reference 2MO αOX40-treated mice (Fig. 6).

## Discussion

Our results demonstrate the ability of caloric restriction (CR) to support immune responses against tumors and effectively maintain CD4 T cell priming during aging. Long-term CR has previously been established to enhance immunity against certain pathogens during aging [35] and the data presented here extend CR-mediated immune enhancement to include increasing tumor immune responses mediated by an immunotherapy. In contrast, a second dietary intervention considered to mimic CR, resveratrol (RES) failed to significantly affect these tumor immune responses following long-term dietary supplementation in aged mice. Unlike RES, CR was observed to maintain priming and activation of antigen-specific CD4 T cells, at levels comparable to young reference animals in the context of αOX40. It is well understood that adaptive T cell-mediated immune responses are critical for effective and durable tumor rejection. Our results suggest the effect of CR on enhancing anti-tumor responses in aged hosts appears to be due in part to the maintenance of the priming environment during aging, which facilitates CD4 T cell activation in the aged hosts following immune stimulation.

It was surprising that the expansion of antigen-activated CD8 T cells primed in aged CR mice was not significantly different from cells primed in ad libitum controls, yet aged CR mice exhibited substantial tumor clearance following αOX40 treatment. CD8 T cells are considered the primary mediators of tumor destruction and are the focus of a number of immune therapies. In addition, CD8 responses have been shown to be responsive to OX40 stimulation [36, 37] and both CD4 and CD8 T cells are needed for αOX40-mediated tumor immunity [34]. The disparity in antigen-specific T cell priming (CD4 > CD8) in CR mice suggests CR maintains the function of a specific subset of antigen presenting cells that prime CD4 T cells. Various DC subsets have been shown to have differential T cell priming capacities. For instance, lymphoid resident CD8^+^ DCs are considered the primary activators of CD8 T cells, via cross-presentation [38], and DCs that migrate from the skin have shown a preference to activate CD4 T cells [39]. This migratory DC subset and other DC subsets, like inflammatory monocyte derived DCs [40], are potent producers of IL-12, a critical cytokine for the activation of CD4 T cells. IL-12 plays a key role in the activation of Th1 CD4 T cells, and we have previously demonstrated that the addition of exogenous IL-12 can partially restore CD4 T cell responses and tumor immunity in aged tumor-bearing animal [11]. Thus, it is possible IL-12 producing DC subsets are sensitive to the effects of aging and long-term CR better maintains these DC subsets during aging.

It has been well documented that CR can maintain an organism’s “health-span” and our data further expand this concept into tumor immunity and tumor immunotherapy. It is well established that animals subjected to long-term CR developed fewer tumors than ad libitum fed animals [41]. An underlying reason for this phenomenon could be the maintenance of tumor immune responses. Our studies in aged mice show tumor immune responses induced by a potential immune therapy, αOX40, could be enhanced in aged animals following CR. A previous study has demonstrated that αOX40 can boost anti-tumor immune responses in aged mice [42], which is also shown in Fig. 3c (PD IgG and PD αOX40); however, CR induces a more pronounced effect on tumor free survival in aged αOX40-treated tumor-bearing mice. Other immunotherapies, such as antagonistic antibodies to the checkpoint inhibitor CTLA-4 and tumor vaccines, may also be enhanced by CR in aged host. We have preliminary data that suggest that in mouse models, aging diminishes both the effect of CTLA-4 on the growth of B16 melanoma and the ability of an anthracyclin-based tumor vaccine to produce protective anti-tumor immunity (data not shown). CR could maintain tumor immunity to boost the effectiveness of these two types of immunotherapies in mice and potentially humans. Interestingly, it appears that endogenous anti-tumor immune responses are also supported by CR. Control 12-month-old CR tumor-bearing animals (IgG treated animals) experienced a consistent and statistically significant increase in survival (Fig. 1c, d). Looking at the endogenous anti-tumor responses in elderly mice, we failed to observe these effects in older 18-month-old mice (Fig. 2), suggesting these responses wane with age regardless of the CR dietary intervention.

Caloric restriction may affect several biological processes besides the immune system that could account for the increased survival of the tumor-bearing mice. Body mass and fat, which have been shown to be related to specific types of tumor genesis and growth (e.g., breast and colon) [43], are reduced in CR animals. The mass of the CR animals and the ad libitum fed animals on LRD and PD was indeed highly significant (Fig. 1b). We also observed a significant difference in mass between ad libitum animals on different formulation of diet, LRD and PD. Yet there was no difference in the survival of the IgG treated mice between the two groups (data not shown). Additional studies are needed to determine the contribution of body mass and fat on tumor immunity in this model.

Due to difficulties with maintaining CR over the long term, CR mimics like RES could be considered options for maintaining tumor immune protection during aging. In fact, RES has been identified as a candidate for cancer chemoprevention [44] and as a CR mimetic that increases “healthspan” [45]; however, RES supplementation did not maintain anti-tumor immune function in the context of αOX40 treatment. RES had a minor effect on the activation of CD4 T cells, but it was four- to fivefold less than young reference mice (2MO) and more importantly had no significant effect on tumor growth and clearance. In contrast, T cell responses in CR mice were similar to young mice. A potential explanation for the discrepancy between RES and CR could be the bioavailability of RES. Greater than 50 % of RES is bioavailable in rodents and humans shortly after ingestion, but RES then undergoes rapid metabolism in the liver, as well as enterohepatic cycling where the metabolized RES is reabsorbed in the intestine further diminishing concentrations in the blood [46, 47]. Levels of RES were not measured in these experiments, but as Bauer et al. showed the dose of RES administered in the diet can produce significant biological effects during aging. However, our findings may suggest that higher doses of RES are needed to sustain immune responses; thus, additional experiments are required to fully determine whether RES can maintain immunity during aging.

These findings have important theoretical implications for optimizing the effect of immune-based cancer therapies in elderly patients and for cancer prevention. CR or possibly an effective mimic initiated when immune responses retain the capacity to regress tumors could preserve endogenous tumor immune surveillance and increase the effectiveness of tumor immunotherapies, such as vaccines and an αOX40. In conclusion, our results show CR maintains immune responses against tumors in aged mice following the stimulation of OX40, a likely target for tumor immunotherapy, and suggest the aged host environment is a critical contributor to immune deficiency during aging.

### Electronic supplementary material

Below is the link to the electronic supplementary material.

Supplementary material 1 (PDF 450 kb)

## Abbreviations

αOX40: OX40 agonist
ACT: Adoptive cell therapy
AL: Ad libitum
CR: Caloric restriction
LRD: Laboratory rodent diet
OVA: Ovalbumin
PD: Purified diet
TCR: T cell receptor
