# Supplementary material for: Caloric restriction maintains OX40 agonist-mediated tumor immunity and CD4 T cell priming during aging
Source: Cancer Immunol Immunother. 2014 Mar 30;63(6):615–26. doi: 10.1007/s00262-014-1542-y (PMC4024137; doi:10.1007/s00262-014-1542-y)
Supplement: Supplementary file 1 — Supplementary material 1 (PDF 450 kb) [file 262_2014_1542_MOESM1_ESM.pdf]

SUPPLEMENTAL FIGURE 1

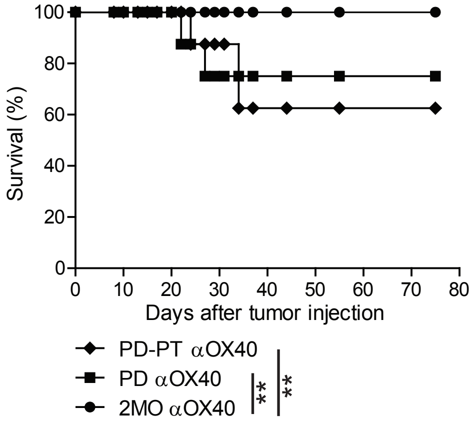

Dietary supplementation with pterostilbene (PD-PT) did not significantly increase αOX40-mediated tumor free survival in aged mice. Two month old and 12 month old female Balb/c mice on a purified diet (PD) or a purified diet supplemented with 1 mg/kg pterostilbene (PD-PT) were injected s.c. with 1 X 10^5 EMT6 tumor cells. Three and seven days later 200 μg αOX40 was injected i.p. and mice were monitored for tumor growth. Data are representative of two independent experiments with n=5-10 mice per experimental group.

SUPPLEMENTAL FIGURE 2

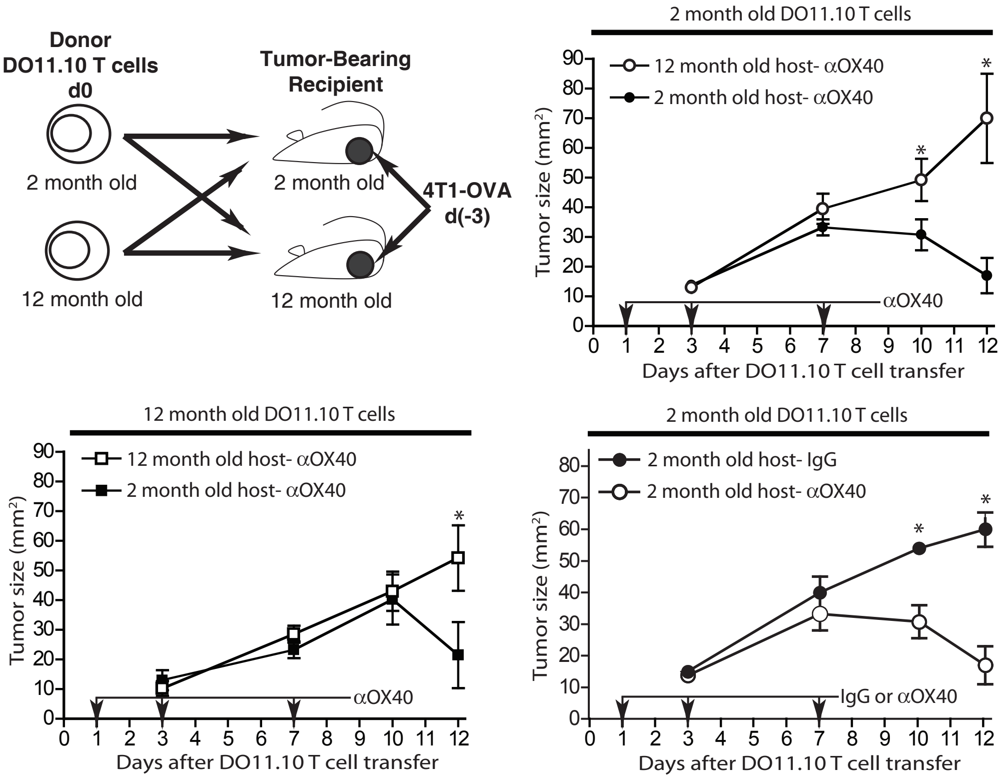

The effects of aging on αOX40-mediated tumor-specific immune responses. Female Balb/c mice ages 2- and 12-months old (AL-LRD) were transplanted s.c. with 5 X 10^5 ovalbumin-transduced 4T1 (4T1-OVA) tumor cells d(-3). CD4 T cells (2 X 10^6) from two- or 12-month old donor DO11.10 mice were adoptively transferred i.v. into two or 12-month old 4T1-OVA tumor-bearing recipient mice (see schematic cartoon) three days after tumor transplantation. Rat IgG or αOX40 (250 μg) was injected i.p. one, three and seven days after adoptive transfer, as identified by the arrows. Mice were then monitored for tumor growth for 12 days after adoptive transfer.
